# Utility of Big Data in Predicting Short-Term Blood Glucose Levels in Type 1 Diabetes Mellitus Through Machine Learning Techniques

**DOI:** 10.3390/s19204482

**Published:** 2019-10-16

**Authors:** Ignacio Rodríguez-Rodríguez, Ioannis Chatzigiannakis, José-Víctor Rodríguez, Marianna Maranghi, Michele Gentili, Miguel-Ángel Zamora-Izquierdo

**Affiliations:** 1 Facultad de Informática, Departamento de Ingeniería de la Información y las Comunicaciones, Universidad de Murcia, 30100 Murcia, Spain; mzamora@um.es; 2 Dipartimento di Ingegneria Informatica Automatica e Gestionale ‘Antonio Ruberti’, Sapienza Università di Roma, 00185 Roma, Italy; ichatz@diag.uniroma1.it (I.C.); marianna.maranghi@uniroma1.it (M.M.); gentili@diag.uniroma1.it (M.G.); 3 Departamento de Tecnologías de la Información y las Comunicaciones, Universidad Politécnica de Cartagena; 30202 Cartagena, Spain; jvictor.rodriguez@upct.es

**Keywords:** continuous glucose monitoring, wearable devices, short-term prediction, univariate time series, machine learning, experimental evaluation

## Abstract

Machine learning techniques combined with wearable electronics can deliver accurate short-term blood glucose level prediction models. These models can learn personalized glucose–insulin dynamics based on the sensor data collected by monitoring several aspects of the physiological condition and daily activity of an individual. Until now, the prevalent approach for developing data-driven prediction models was to collect as much data as possible to help physicians and patients optimally adjust therapy. The objective of this work was to investigate the minimum data variety, volume, and velocity required to create accurate person-centric short-term prediction models. We developed a series of these models using different machine learning time series forecasting techniques suitable for execution within a wearable processor. We conducted an extensive passive patient monitoring study in real-world conditions to build an appropriate data set. The study involved a subset of type 1 diabetic subjects wearing a flash glucose monitoring system. We comparatively and quantitatively evaluated the performance of the developed data-driven prediction models and the corresponding machine learning techniques. Our results indicate that very accurate short-term prediction can be achieved by only monitoring interstitial glucose data over a very short time period and using a low sampling frequency. The models developed can predict glucose levels within a 15-min horizon with an average error as low as 15.43 mg/dL using only 24 historic values collected within a period of sex hours, and by increasing the sampling frequency to include 72 values, the average error is reduced to 10.15 mg/dL. Our prediction models are suitable for execution within a wearable device, requiring the minimum hardware requirements while at simultaneously achieving very high prediction accuracy.

## 1. Introduction

Type 1 diabetes mellitus (DM1) is a disease characterized by high blood sugar levels that result from the body’s inability to produce insulin. In a healthy person, glucose homeostasis is a closed-loop system that is able to regulate blood glucose levels [1]. The pancreas houses the β cells, which are sensitive to high glucose levels and produce insulin, a strong hormone able to reduce hyperglycemia. This regulation is not possible in DM1. This is an autoimmune disease in which the body destroys the insulin-producing cells in the pancreas, and is the more aggressive form of the disease. Patients with DM1 do not produce any insulin and must inject this hormone exogenously or wear an insulin pump to reduce their glucose levels [2]. Therefore, the objectives of diabetes management include maintaining homeostasis and blood glucose near normal levels, and avoiding hypoglycemia and ketoacidosis as well as other long-term complications (e.g., cardiovascular diseases, etc.) [3].

Diabetic people need to establish near-normoglycemia by frequently performing capillary blood glucose monitoring, although with continuous glucose monitoring (CGM) devices such as the Dexcom G6 (Dexcom Inc., San Diego, CA, USA), this type of self-monitoring is no longer needed. These groundbreaking CGM devices can be used effectively for lowering HbA1c (Hemoglobin A1C), reaching target HbA1c and reducing glucose variability [4].

However, patients also need to consider their daily activities and diet to determine their daily insulin requirements [5]. Essentially, they continuously mentally predict the future evolution of blood glucose levels and then decide, following medical staff advice, when and how much insulin is required to maintain metabolic control and avoid eventual hyperglycemia or hypoglycemia that triggers subsequent hyperglycemia. The important task of accurately forecasting future blood glucose levels to infer insulin dosages is complicated. A patient must consider several influential factors that add to the problems with their subjective assessment, which can lead to mistakes.

Fortunately, technologies available for DM1 are becoming more sophisticated. In addition to CGM, advanced insulin pumps include low-glucose suspension and predictive low-glucose suspension (PLGS), which can prevent episodes of hypoglycemia. They also include automated delivery methods such as hybrid closed-loop (HCL) systems. Messer et al. provided an updated view of this subject [6]. These systems increase the time within normal range by minimizing hypoglycemia and hyperglycemia. Commercial availability of automated insulin delivery systems is currently limited, but patient access to these systems is anticipated to improve in the near future [7]. Telemedicine completes the paradigm; patients receive care from a specialist remotely assisting to improve glycemic control.

Wearable electronics could be added to this management platform, offering a completely new dimension in diabetes management, and the addition of features could improve glucose level forecasting. Numerous wearable devices have the potential to be introduced into a DM1 management system to monitor, in addition to the CGM monitoring of glucose levels, different physiological aspects of diabetic people, such as interstitial/blood glucose levels, exercise and activity, heart rate, electrocardiogram, and others. Wearable devices broadly available on the market deliver multiple measurements per minute of the physiological condition and the daily activity of an individual. A complete characterization of a person with diabetes could produce a large amount of data [8], although some features (such as weighing food) still need to be accomplished by the user, or they rely on a subjective factor (such as emotional state), leading to some inaccuracies or mistakes. These data could potentially help to better predict the magnitude, probability, frequency, and duration of fluctuations in glucose levels [9]. These predictions, when combined with reliable insulin pumps, can realize the vision of closed-loop control of blood glucose, also known as the artificial pancreas [10].

Along with the aforementioned benefits, the use of multiple wearable devices necessitates their daily maintenance, which increases the complexity of the lives of diabetic people [11], potentially leading to a decline in the perceived quality of life [12]. The wireless and personal nature of wearable sensors also exposes them to a number of unique security vulnerabilities with direct consequences on the confidentiality of private data. Therefore, it is important to understand the benefits of creating big data sets, the greater computational efforts required to process the data, the costs to the users for collecting the data, and the repercussions for misusing the data.

In this article, we introduce a discussion on predicting glucose levels (GL) to regulate metabolic control without using large volumes of data, collected only from CGM. We examine the accuracy of forecasting future GL when the variability, volume, and velocity of the data collected from the CGM devices are minimized; these are three critical components for characterizing big data [13]. In particular, we examine the accuracy that can be achieved in predicting GL by (a) minimizing variability using only one aspect of the individual’s physiological condition and daily activity from the CGM data; (b) minimizing volume of data by limiting the historical data to three hours (from 36 h); and (c) minimizing velocity of data by increasing the sampling rate from one value every 5 min to one value every 15 min. We provide a scenario in which diabetic people wear only one medical device, minimizing possible overhead in their daily lives, and minimizing collected data. Therefore, we reduce the disadvantages associated with data collection and thus potentially increase end-user acceptance.

## 2. Aim

The aim of this study was to design automatic GL prediction models for type 1 diabetes mellitus that are suitable for wearable processors with limited computational and storage capabilities. We aimed to evaluate different machine learning methods in developing accurate univariate time series prediction using a comprehensive CGM database. Methodology improvements on previous approaches include:

- Designing automatic blood glucose level prediction models using only CGM data;
- Assessing the accuracy of three different machine learning methods for univariate time series prediction;
- Conducting a detailed passive monitoring campaign involving 25 DM1 patients for up to 14 days in real-world conditions;
- Collecting a comprehensive data set that can be used to evaluate the performance of different prediction models;
- Comparing the accuracy of the three prediction models based on the blood glucose time series obtained from the study;
- Evaluating the effect of the volume of historical data for improving the accuracy of the prediction models;
- Evaluating the effect of the velocity of CGM sampling for improving the accuracy of the prediction models;
- Identifying the minimum data variety, volume, and velocity necessary to achieve high prediction accuracy.

After the review of previously published work in Section 3 and discussing significant differences with the current results, the patient monitoring system and study design are presented in Section 4. The methodology adopted for developing the patient prediction models is also described in detail in this section. The results of the prediction performance of the different patient prediction models developed are presented in Section 5. Our conclusions are drawn in Section 6.

## 3. Relevant Literature

Numerous attempts have been made to develop models for predicting blood glucose levels in diabetic people. Today, the most well-studied approach for blood glucose prediction is based on detailed physiological models that try to capture the dynamics of glucose-relevant variables within different systems in the body. Most physiological models categorize the overall dynamics into three compartments: meal absorption, insulin, and glucose dynamics. This results in a series of equations that can be solved analytically [14] and, in some cases, are equivalent to classical proportional-integral-derivative (PID) models [15,16]. These models are comprised of multiple equations and require hardware processors that cannot be easily supported by wearable devices. In addition, the large number of parameters used to describe the multiple equations complicates their adjustment tune for individual patients. Some researchers have attempted to reformulate these models using multiparametric programming techniques so that they require less computational power [17]. Still, using the existing wearable processors, providing a feasible execution environment to support the computation of these models within a wearable device is challenging.

More recently, a new approach has been proposed to address this problem by applying machine learning techniques. The key concept behind this approach is that repetitive cycles exist in glucose–insulin dynamics, e.g., before/after meals, before/after bedtime, and predictable insulin sensitivity changes throughout the day due to circadian variations in hormone levels. Studies have evaluated patterns by time of day, even showing variability according to the day of the week [18]. Several data-driven models have been developed to explore the repetitive nature of glucose–insulin dynamics, using various techniques for time series forecasting such as autoregressive (AR), impulse-response (IR), autoregressive exogenous input (ARX), autoregressive moving average exogenous input (ARMAX), and autoregressive integrated moving average (ARIMA). For example, Estrada et al. developed an ARX model that relies on blood glucose levels and insulin dosages to forecast future blood glucose levels within a 45-min prediction horizon [19]. Other approaches, as performed by Nuryani et al., explored the use of support vector machines (SVMs) to predict hypoglycemia using electrocardiograms (ECG) in addition to blood glucose levels and insulin injections [20]. Marling et al. used SVM models, combining data gathered from wearable activity trackers, monitoring heart rate, galvanic skin response, and skin/air temperatures [21]. As these models rely on a diverse data set, even under comparable evaluation conditions, it is hard to understand the benefit of including each different type of data within the prediction model. Since some of the data are created by humans, e.g., meal information, we also need to consider that these data can be affected by subjectivity and human error. Inevitably, under such conditions, we cannot complete a reliable comparative evaluation of their predictive accuracy.

Another class of empirical models uses artificial neural networks (ANNs) to learn the relationship between past and future blood glucose levels and also consider other data sources. For example, Pappada et al. developed an approach that combines the fingerstick method to measure blood glucose levels with insulin dosages, meals, and some lifestyle conditions and emotions [22]. Zecchin et al. developed a predictor based on a neural network (NN) model and a first-order polynomial extrapolation algorithm that combines past CGM sensor readings with data on carbohydrate intake [23]. Unfortunately, how the techniques have been applied to develop the prediction models, as well as the different pre-processing signal processing conducted, is difficult to reproduce. NN-based models require a large volume of data to properly calculate the internal parameters of the networks and avoid overfitting. However, the majority of these studies assessed the performance of the models either using simulated datasets or real-world data collection campaigns of very limited duration. It is therefore important to ensure that data used for the training of these empirical model is, by design, long-term and involves real individuals.

Many examples of glucose forecasting exist in some form that include past glucose values but also use other features such as insulin regimen, meals, exercise, etc. The possibility of forecasting using only past glucose values has been studied, and this could provide some benefits: relying only on one device (CGM), avoiding human error due to subjectivity, and simplifying the calculation process of the algorithm. This leads to an interesting possibility: applying this algorithm in a constrained device, such as a smartphone, and then eliminating the risks of completing this key step in the cloud, which is susceptible to loss of cellular signal in remote areas or to general failure in communications. Forecasting locally could supplement cloud computing and be activated in case of emergency.

The concept of developing data-centric prediction models relying solely on CGM technologies has been studied in the past [24,25,26]. Different machine learning techniques have been used to develop data-centric prediction models that can be used for early hypoglycemic/hyperglycemic alarms and for closing the glucose regulation loop with an insulin pump. For example, Sparacino et al. evaluated an AR-based model within a hospital environment involving 28 DM1 patients for 48 h [24]. Eren-Oruklu et al. developed a model using ARIMA based on data collected from monitoring 22 DM1 patients for 48 h [25]. Hamdi et al. monitored 12 patients to develop a model combining support vector regression and a differential evolution algorithm [26]. In contrast with our work, the performance of these approaches was measured within a carefully designed hospital environment with rigorously controlled conditions. As a result, it is not evident how the results could be extrapolated for a daily routine. More importantly, the data collection campaigns conducted to evaluate the performance of the proposed models were conducted for a very short period of time, and they used different sampling rates, resulting in data sets of diverse volumes and velocity. It is therefore almost impossible to directly and quantitatively compare the different machine learning techniques or even the specific patient-centric models.

In this work, we conducted an extensive passive patient monitoring study in real-world conditions and not in a controlled hospital environment. The study involved significantly more patients and a longer duration when compared to previous relevant studies, thus providing a basis for comparing the different techniques.

To the best of our knowledge, a proper and complete comparison between glucose prediction algorithms has not been previously completed. At best, in some works, a few methods were compared using glycemia but also (depending on the paper) a few other factors assuming some parameters, and it is not possible to extract overall conclusions. The data origins were different from one study to another, so developing a comparison among the methods studied in different works becomes a purely subjective task.

Random forest (RF) and SVM were used and compared [27] (as well as in decision trees and the naïve Bayes models) to predict hypoglycemia using glycemia and medication as factors in an undetermined patients data set. RF and SVM were the best at predicting low levels of blood glucose, with up to 97.5% and 97% accuracy, respectively, in a one-day window. The results of the other two models were significantly less accurate. Unfortunately, the work only focused on hypoglycemia and cannot be generalized. SVM and RF were also found to be more successful tools for predicting the long-term evolution of DM1 compared with other models [28] with a wide set of predictive factors related to the long-term status of patients, the course of the disease, and its aggravation. Although the study was targeted to diagnosis, it provides a good example of the good performance of these machine learning (ML) algorithms in tasks related to diabetes. Data were obtained from a repository. RF was also used to diagnose DM1 by Xu [29] from a data set from a public hospital, obtaining the best performance (85% success) in comparison with other methods; the ID3 (Iterative Dichotomiser 3) algorithm, the naïve Bayes algorithm, and the AdaBoost algorithms achieved accuracies of 78.57%, 79.89%, and 84.19%, respectively. In another comparison study, Bunescu et al. [30] chose SVM as an approach to predict glycemia, and compared it with ARIMA. In this case, SVM included insulin dosages, and produced better performance than the autoregression, with a root mean square error (RMSE) of 22.2 mg/dL at 30 min and 41.3 mg/dL at a prediction horizon (PH) of 60 min. The results for ARIMA were slightly worse.

For the first time, we conducted a comparative evaluation of different machine learning techniques over a common dataset, thus providing a solid quantitative performance comparison.

## 4. Research Design and Methods

### 4.1. Patient Monitoring System

The patient monitoring system consisted of a wearable Abbott Freestyle Libre (Abbott Laboratories, Chicago, IL, USA), which is a flash glucose monitoring (FGM) system combined with a smartphone to transmit data to a central server for processing. The FGM sensor includes local memory that is capable of storing the past measurements for up to 8 h. Software installed on the patient’s device (either a smartphone or tablet) periodically connects to the FGM device using a secure, short-range wireless connection following the NFC (Near Field Communication) standard to collect the most recent measurements. The data are then securely forwarded to a centralized database where they are streamlined for further processing by the prediction models. More information on the ICT system used to collect the data is presented in [31].

The FGM sensor can provide measurement of blood glucose (mg/dL) every minute [32] using a sensor inserted under the skin. The value of the FGM sensor is therefore an estimate of the actual glucose value present in the bloodstream with a mean absolute relative difference (MARD) of 11.4% [33] as stated by the manufacturer. The sensitivity range of the sensor is 40 mg/dL (below which the sensor only indicates “low”) to 500 mg/dL (above which the sensor only indicates “high”). The measurements experience a delay of some minutes. For this reason, a lag time of about 5 to 10 min occurs between the data provided by the FGM sensor and the real glucose level in the bloodstream of the patient [34]. Mathematical methods can suitably compensate for the lack of accuracy due to this delay [35], reducing it to just six minutes. When the FGM sensor is applied, the calibration period is only a few hours [36]. The FGM sensor has a maximum life of 14 days. In total, 5400 h of data were collected.

### 4.2. Study Design

We conducted a study using the above system involving 25 DM1 patients with diabetes during 2018 under the supervision of the Endocrinology Departments of the Morales Meseguer and Virgen de la Arrixaca Hospitals, in the city of Murcia (Spain). The study was conducted in accordance with the Helsinki Declaration. The study was approved by the Ethics Committee of Universidad de Murcia, Spain. Data storage complied with the stricter data protection rules for protecting personal information. The clinical characteristics of the patients considered in the study are summarized in Table 1. All participants were fully informed about the purpose of the experiment and provided written informed consent and assent according to the national regulations. The participants were informed about the data collected during the study and how they were stored.

Participants were recruited via advertisements and word of mouth. The group was composed of 14 men and 11 women, all of them under medical treatment and professional supervision. Patients were students or office workers of the Universidad de Murcia, 18 to 56 years of age (average 24.51); most of them were young adults. Patients were chosen with an illness duration of at least 5 years to guarantee familiarity with the course of the disease and all of them were familiarized with the use of the Abbott Freestyle Libre FGM sensor (Abbott Laboratories, Chicago, IL, USA). All users had on-demand access to the FGM data to use that information to improve their glucose control. Routine downloading of diabetes devices (blood glucose monitors, pumps, or CGM) is associated with better glycemic control.

All patients reported leading a healthy life, and all practiced sports at least 3 times per week. Their schedules were controlled, ensuring that all of them followed reasonably well-regulated daily routines without abrupt changes in their daily timetables. Patients’ DM1 was usually well controlled, all having glycated hemoglobin (HbA1c) values between 6% and 7% at the beginning of the experiment.

During the passive monitoring period, patients were encouraged to follow their daily routines and apply a balanced diet according to their caloric necessities. All patients were encouraged not to deviate from their doctors’ advice during the monitoring period. All participants followed a basal-bolus regimen, using slow insulin such as Levemir (Novo Nordisk A/S, Bagsværd, Denmark), Tresiba (Novo Nordisk A/S, Bagsværd, Denmark), or Lantus (Sanofi-Aventis Deutschland GmbH, Frankfurt, Germany), which have flat-action curves, and fast insulin such as Humalog Lispro (Lilly Co., Indianapolis, IN, USA). The former provide more than 24 h as a basal coverage, and the latter is used to compensate for a rise in glucose, which can be due to the intake of a meal or hyperglycemia caused by other factors.

### 4.3. Methods for Glucose Level Prediction

Our goal was to develop reliable prediction models that can highly accurately estimate the future glucose level based only on current and past data collected from the FGM sensor. We developed patient-centric models using different machine learning methods to capture the properties of the blood glucose time series of each individual patient. Therefore, for each patient, we generated a separate prediction model.

**Figure 1:**
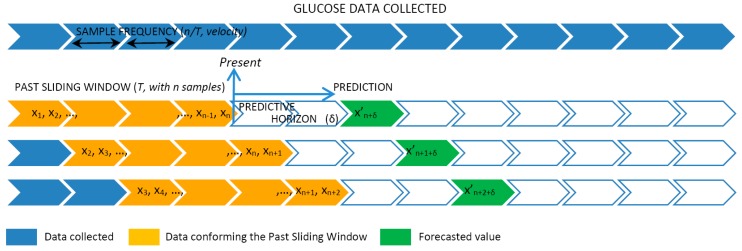
Time series analysis and cross-validation with slide-window.

The data provided by the FGM sensor were sampled with a sample frequency (SF) of 1 measurement every 5, 10, or 15 min. Therefore, the SF controlled the velocity of the data that we were considering. The values sampled were used to create a past sliding window (PSW) including historic values varying from 3 to 36 h. The PSW controlled the volume of the data the model used for the prediction. Given the sliding window data, the model continuously predicted the glucose levels at preset PHs at 15, 30, 45, and 60 min ahead of the present time. Figure 1 graphically represents the fragmentation of the data collected into windows that are used as input for the patient-centric prediction model.

In detail, at a given time *t,* the data collected from the FGM sensor over the past period *T* were used to generate the training set of *n* data points ${\left\{x_{i}\right\}}_{i=1}^{n}$, where *x_i_* ∊ *R* is each individual data point received from the FGM sensor. We say that *n* characterizes the volume of the data set and *n/T* is the velocity of the data. Given this training set, the goal of the prediction model is to approximate the real underlying mapping accurately enough so that the next *δ* value can be predicted, which is the output value *y*(*t* + *δ*) ∊ *R* (this is, the forecasted x′_n + *δ*_) for that time series, where *δ* is the prediction horizon.

The sliding window works as follows. As soon as a new FGM value is received, the training set is reorganized by removing the oldest observation (*x*_1_), shifting all the values 1 position up (i.e., *x_i_* becomes *x*_*i*−1_) and finally adding the new value received as the newest one (*x_n_*). As such, the dataset size and ordering of the observations is always preserved.

The methods considered in this work for glucose level prediction are the following:

1. Autoregressive integrated moving average (ARIMA): The univariate (single vector) ARIMA is a forecasting technique that projects the future values of a series based entirely on its own inertia [37]. ARIMA attempts to characterize the movements in a stationary time series as a function of a combination of autoregressive (AR) integration (I), referring to the reverse process of differencing to produce the forecast, and moving average (MA) operations. The model is stated as ARIMA(p,d,q) representing the order of the autoregressive components (p), the number of differencing operators (d), and the highest order of the moving average term (q).
2. Random forest (RF): RF algorithms employ a technique known as bagging, whereby data instances are resampled multiple times to produce multiple training subsets from the training data [38]. Decision trees are then created from each training subset until ensembles of trees are created. Each tree then casts a unit vote for the outcome of an incoming data instance class label. RF is flexible with constrained computational resources required.
3. Support vector machine (SVM): The basic idea of SVM for time series approximation is mapping the data into a high-dimensional feature space via nonlinear mapping and then performing a linear regression in the feature space [39]. The nonlinear mapping can be efficiently computed through a kernel function without iterating over all the corresponding data points. Given the kernel function, the SVM learner tries to find a hyperplane that separates positive from negative data points and simultaneously maximizes the separation (margin) between them. This method is known to be resilient to overfitting and to have good generalization performance due to the max-margin criterion used during optimization. SVM is guaranteed to converge to a global optimum due to the corresponding convex optimization formulation.
4. Prediction performance measure: The performance of each different model is evaluated in relation to the data volume (*n*), data velocity (*n/T*), and the prediction horizon (*δ*). The values predicted by each patient-centric model are compared with the actual values collected from the FGM sensor. The RMSE is used to evaluate the prediction accuracy, as this is the most broadly used metric in the relevant literature to measure prediction performance as reported in Section 3. The RMSE represents the square root of the differences between predicted values and observed values.
5. Implementation details: The initial models were implemented using the programming language R v3.5.0 (R Foundation for Statistical Computing, Vienna, Austria) in combination with the CARET (Classification And REgression Training) library v6.0-84. For the evaluation of the performance, an AMD Ryzen 7 1700X processor (Advanced Micro Devices, Inc., Santa Clara, CA, USA) was used, operating at 3.8 GHz with 32 GB DDR4 RAM at 2666 MHz CL19.

## 5. Results

**Figure 2:**
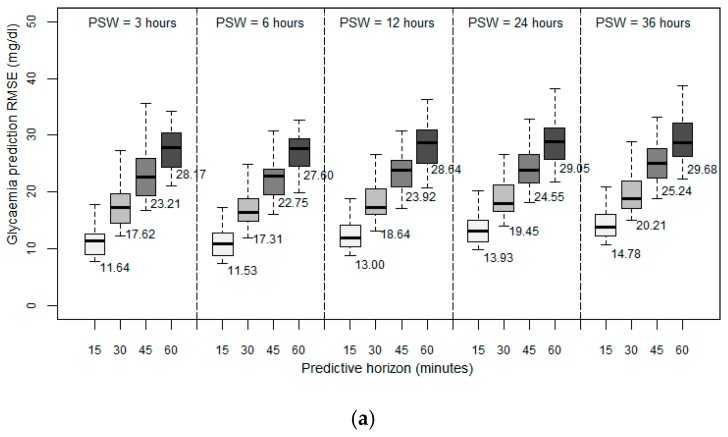

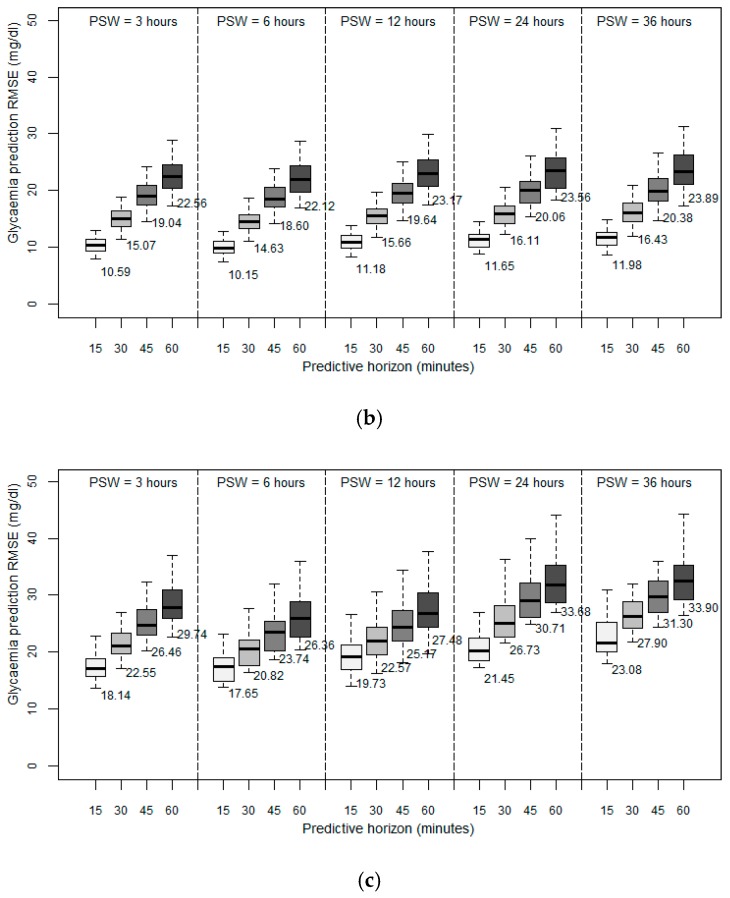
Root mean square error (RMSE in mg/dL) with fixed sampling frequency: (**a**) Autoregressive integrated moving average (ARIMA), (**b**) random forest (RF), and (**c**) support vector machine (SVM) with past sliding window sizes (PSWs) of 3, 6, 12, 24, 36 h; predictive horizons (PHs) of 15, 30, 45, and 60 min; and sampling frequency (SF) of 5 min.

We first examined the performance achieved in predicting future blood glucose levels when we only used historical data on the blood glucose levels. Figure 2 depicts the results obtained in terms of RMSE between the predicted values and the observed values. We first observed that for all the patient models developed, the prediction error increases as the PH increases, which seems reasonable since the data collected are progressively further from the prediction. In general, for the three different methods used to develop the patient models, acceptable predictions were achieved for PHs of 15 and 30 min, with average RMSEs lower than 20 mg/dL for ARIMA (Figure 2a) and RF (Figure 2b), and lower than 28 mg/dL for SVM (Figure 2c), the further PHs being sufficient to demonstrate the trend in the glucose.

We then analyzed the importance of historical data volume on the prediction accuracy of future glycemic levels. The window size for the past data allows the model to capture the temporal structure in the time series. We expected that increasing the volume of historical data would improve the prediction accuracy. For this reason, we designed the first experiments by training the patient models using different past sliding window (PSW) sizes that contained the observations of the past 3, 6, 12, 24, and 36 h. In this experiment, the data velocity was fixed to one sample every five minutes and a PH of 15, 30, 45, and 60 min.

Investigating the volume of historical data needed to provide an accurate prediction, as expressed by the different PSW sizes used, the results achieved indicated that all the developed patient models have a reduced RMSE with a window size of six hours. Initially, when we increased the volume of historical data from three to six hours, we observed a positive effect on the prediction accuracy. However, when we increased the window size further, we observed a negative effect on the overall performance. A window size of 12 h resulted in an RMSE higher than that achieved with a window size of three hours. Any further increase in the volume of historical data further deteriorated the achieved accuracy.

Experimental evidence indicated that regardless of the method used to develop the models, and for all the prediction horizons, a backward limit (six hours) exists when considering past data to improve accuracy of the prediction; so an optimum point beyond which past data stop being significant and introduce more error into the forecasting. Previous works introduced this order of magnitude, linking the idea of circadian cycles [40] and the slots of morning, afternoon, and night.

Examining the performance achieved by the three different methods used to develop the patient models, the results indicated that the RF method is the most accurate, producing better predictions with all the PSWs and for all the PHs considered. RF also presented less standard deviation, which means that it is more independent of the specific characteristics of each patient. We think that particular routines and the peculiarities of each subject affect accuracy.

**Figure 3:**
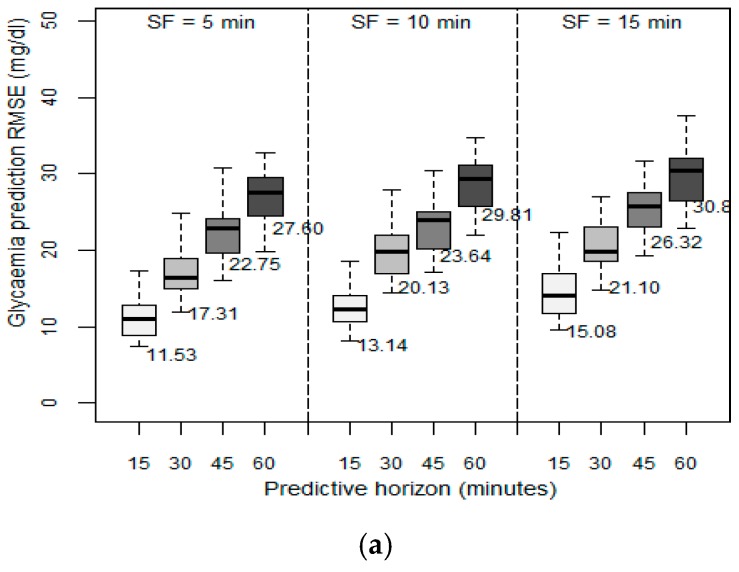

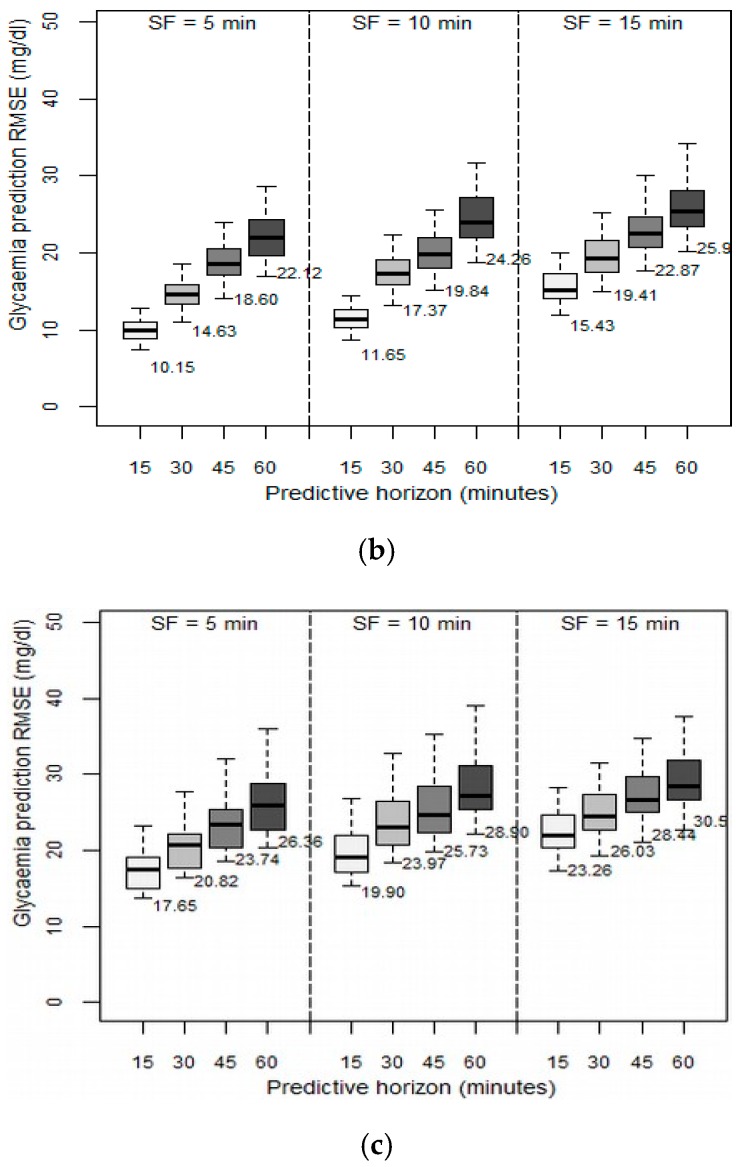
Root mean square error (RMSE in mg/dL) with fixed past sliding window (PSW) of 6 h, SFs of 5, 10, and 15 min; and PHs: 15, 30, 45, and 60 min for (**a**) ARIMA, (**b**), RF, and (**c**) SVM.

We then examined the effect of the velocity of the data collected from the GCM sensor on the prediction ability of the patient models. In the previous set of experiments, the data velocity, (i.e., the SF), was set to one sample every five minutes. In this set of experiments, we evaluated the effect of using different sampling frequencies between 5 and 15 min on the achieved RMSE. Figure 3 depicts the results obtained in terms of RMSE between the predicted values and the observed values when the past sliding window was fixed to six hours, as this was the optimum value identified in the previous experiment. The higher the SF, the higher the resulting RMSE, regardless of the PH. However, as the PH increased, the SF effect is greater: with a PH of 15 min, when increasing the SF to 10 min, RMSE increased by 50%, but with the same change in SF, in a PH of 60 min, RMSE increased only around 10% to 12%. We concluded that the decision to reduce SF depends on the desired future prediction length. By only changing SF from 5 to 10 min, acceptable RMSEs were still achieved. We observed that the patient models that are based on the SVM method reached very high RMSEs when SF was 15 min. We therefore concluded that models based on the SVM method should use an SF of five minutes.

As with the previous experiment, we again observed that the patient models developed using the RF method achieve a better performance for each different SF considered. RF also has a lower standard deviation, so it is more independent of the variability of the data, and hence of each patient’s characteristics.

## 6. Conclusions

In DM1 patients, glucose dynamics are influenced by insulin reactions, diet, lifestyle, etc., and are characterized by instability and nonlinearity [41]. Therefore, understanding how much data on the past physiological conditions of the individuals is needed to achieve an accurate forecast is essential. A common approach followed in the literature regarding prediction models is to collect as much data as possible in an attempt to achieve near-normal control. In this study, we worked in the opposite direction: we investigated the minimum variety, volume, and velocity needed to accurately predict future levels of blood glucose in individuals with diabetes.

We comprehensively passively monitored 25 individuals for up to 14 days. The obtained data set is novel, since to the best of our knowledge, this is the first time that CGM data have been registered for such a large number of people and with a long duration in real-world conditions. The data allowed us to conduct a detailed comparative evaluation of prediction models developed using three different machine learning methods.

Our evaluation indicates that using only one type of data, that of past blood glucose levels, allowed the prediction of future glucose levels with a very low average error of 10.15 mg/dL with a 15 min prediction horizon; for a 60 min horizon, the average error was as low as 22.12 mg/dL. In this setting, the volume of data is restricted to six hours and the velocity to one sample every fie minutes. In other words, only 72 historical values are required to achieve very good performance. If we further reduce the velocity to one sample every 15 min, that is, using only 24 historic values, the achieved accuracy is still within an acceptable error level of 15.43 mg/dL for a 15 min prediction horizon and 25.9 mg/dL for a 60 min horizon.

Our study demonstrates that a volume of historical data over six hours produces optimal prediction accuracy. Any further increase in the data volume does not further improve accuracy. All developed prediction models performed worse with a large volume of historical data for all different prediction horizons investigated. Finally, our work shows that the glucose prediction models developed using the RF method are the most accurate and produce the best performance.

In this sense, we provided concrete evidence for wearable device manufacturers: (a) they do not need to use large memory units to increase the volume of the stored data and (b) they do not need to use high frequency for collecting data from the sensors. As such, the hardware requirements and the power consumption of the next-generation wearable devices can be reduced while delivering products of (a) smaller size, thus less weight, and (b) a longer lifespan, thus requiring less maintenance from the end users. Therefore, they can deliver new products with an improved quality-of-experience and with a potentially higher user acceptance.

For future work, our monitoring campaign should be extended beyond 10 days and the effect of the veracity of data collected should be confirmed by examining the possibility of missing data, e.g., due to user error or low-quality sensing devices, on the accuracy of the prediction models. Prediction models are providing a forecast of the precise blood glucose level given a prediction horizon. We are interested in developing models that predict a trend for a much larger prediction horizon, e.g., four to six hours, especially during periods of extreme values, e.g., below 70 or above 250 mg/dL. Finally, we wish to extend our measurement campaign by involving patients with type 2 diabetes and sensor devices from different manufacturers.

## Figures and Tables

**Table 1:** Data regarding the patients considered in the study.

Feature	Value		
Subjects (number)	25		
Sex	14 men, 11 women		
Occupation	16 students, 9 office workers		
Population Feature	Median	Min	Max
Age (years)	24.51	18	56
Body Mass Index (BMI, kg/m^2^)	22.20	19.42	24.80
Duration of diabetes (years)	9	5	29
Fingersticks per day	7	5	12
Insulin units per day (fast insulin + slow insulin, median)	47	36	59
HbA1C (%)	6.8	6.3	7.8
